# Microscopic basis for the band engineering of Mo_1−x_W_x_S_2_-based heterojunction

**DOI:** 10.1038/srep14808

**Published:** 2015-10-07

**Authors:** Shoji Yoshida, Yu Kobayashi, Ryuji Sakurada, Shohei Mori, Yasumitsu Miyata, Hiroyuki Mogi, Tomoki Koyama, Osamu Takeuchi, Hidemi Shigekawa

**Affiliations:** 1 Faculty of Pure and Applied Sciences, University of Tsukuba, Tsukuba 305-8573, Japan; 2 Department of Physics, Tokyo Metropolitan University, Hachioji, Tokyo 192-0397, Japan; 3 JST-PRESTO, Kawaguchi, 332-0012, Japan

## Abstract

Transition-metal dichalcogenide layered materials, consisting of a transition-metal atomic layer sandwiched by two chalcogen atomic layers, have been attracting considerable attention because of their desirable physical properties for semiconductor devices, and a wide variety of pn junctions, which are essential building blocks for electronic and optoelectronic devices, have been realized using these atomically thin structures. Engineering the electronic/optical properties of semiconductors by using such heterojunctions has been a central concept in semiconductor science and technology. Here, we report the first scanning tunneling microscopy/spectroscopy (STM/STS) study on the electronic structures of a monolayer WS_2_/Mo_1−x_W_x_S_2_ heterojunction that provides a tunable band alignment. The atomically modulated spatial variation in such electronic structures, i.e., a microscopic basis for the band structure of a WS_2_/Mo_1−x_W_x_S_2_ heterojunction, was directly observed. The macroscopic band structure of Mo_1−x_W_x_S_2_ alloy was well reproduced by the STS spectra averaged over the surface. An electric field of as high as 80 × 10^6^ Vm^−1^ was observed at the interface for the alloy with x = 0.3, verifying the efficient separation of photoexcited carriers at the interface.

Monolayer transition-metal dichalcogenides (TMDs), consisting of a transition-metal atomic layer (e.g., W and Mo) sandwiched by two chalcogen atomic layers (S, Se and Te), have been attracting considerable attention because of their desirable physical properties for semiconductor devices such as a high optical absorption coefficient^1^,^2^,^3^, efficient photoluminescence^4^,^5^, and a valley structure^6^,^7^,^8^. Different combinations of transition metals and chalcogen atoms can provide band gaps varying over a wide range (1–3 eV), and a wide variety of pn junctions, which are essential building blocks for electronic and optoelectronic devices, have been realized using such atomically thin materials^9^,^10^,^11^,^12^,^13^,^14^,^15^. The engineering of the electronic/optical properties of semiconductors by using such heterojunctions has been a central concept in semiconductor science and technology, and from a fundamental standpoint, heterostructures formed by two-dimensional materials provide a new platform for exploring new physics.

Recently, tunable band alignment has been realized using Mo_1−x_W_x_S_2_ alloy^16^,^17^,^18^,^19^, showing the high potential of this material for future device technology. However, the microscopic view of the electronic structures of Mo_1−x_W_x_S_2_-based heterojunctions, which is the basis for the band engineering, has not yet been clarified. Here, we report the first scanning tunneling microscopy/spectroscopy (STM/STS) study on the electronic structures of a monolayer WS_2_/Mo_1−x_W_x_S_2_ lateral heterojunction. The atomic-scale analysis by STM/STS allowed the verification of the basic semiconductor physics of this material, providing a microscopic basis for the band engineering.

## Results

### Atomically modulated electronic structures and macroscopic band structure of Mo_1−x_W_x_S_2_ alloy

Monolayer WS_2_/Mo_1−x_W_x_S_2_ lateral heterojunctions for the STM measurements were prepared by chemical vapor deposition (CVD) on a graphite substrate (see Method). All STM/STS measurements were carried out using an Omicron low temperature-STM and a W tip at 87 K.

**Figure 1:**
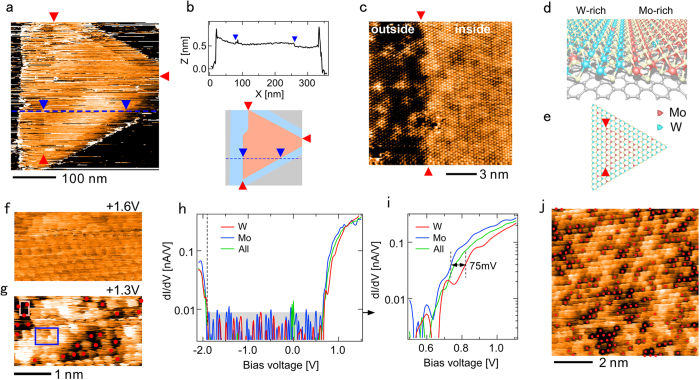
STM/STS on the WS_2_/Mo_1−x_W_x_S_2_ heterostructure. (**a**) STM image of a typical Mo_1−x_W_x_S_2_ structure grown by CVD on a graphite substrate (*V*_s_ = +2.5 V, *I*_t_ = 0.25 A). (**b**) Cross section along the blue line in a and schematic structure of the STM image in (**a**). (**c**) Magnification of the part of the interface indicated by two red triangles at the top and bottom in a. (**d**,**e**) Schematic models of the STM images in (**c**,**a**). (**f**,**g**) STM images of Mo-rich Mo_1−x_W_x_S_2_ area obtained at sample bias voltages of *V*_s_ = +1.6 and +1.3 V, respectively. (**h**) d*I*/d*V* spectra obtained in the Mo and W areas indicated by the white and blue squares in (**g**). The gray rectangle indicates the noise level. (**i**) Magnification of the spectra in (**h**) near the conduction band edge *E*_CBM_. (**j**) High-resolution STM image of Mo-rich Mo_1−x_W_x_S_2_ area.

Figure 1a,b show a typical STM image of Mo_1−x_W_x_S_2_ monolayer alloy with a triangular shape and the cross section along the blue line in the STM image, respectively. The low resolution of the STM image in Fig. 1a is due to the fact that it was difficult to obtain a high-quality image at a low temperature when the observed area included an island edge. As shown in the cross section, the height of the layer is ~0.7 nm over the triangular structure. Close inspection of Fig. 1a and the cross section reveals a slightly smaller triangular area inside the triangular shape, with the interface between the inner and outer triangles shown by two dark blue triangles. The two triangular structures are schematically shown below the cross section in Fig. 1b, in which the three red triangles indicate the positional relationship with Fig. 1a.

Figure 1c shows a magnification of a part of the interface between the smaller inner triangle and the larger outer triangle along the line joining the two red triangles at the top and bottom of Fig. 1a. The interface in the STM image in Fig. 1c is also indicated by two red triangles. To obtain the Mo and W distributions, the bias voltage was set at +1.35 V and the Mo and W atoms were imaged with a suitable contrast. As shown in Fig. 1c, small bright islands are distributed in the outer area (left side of the image), while dark lines form a netlike structure in the inner area (right side of the image), as previously observed for Mo_1−x_W_x_S_2_ alloy exfoliated from its bulk structure^17^. Namely, the inner triangular area in Fig. 1a (right side of Fig. 1c) is not a second layer but a Mo-rich Mo_1−x_W_x_S_2_ alloy area surrounded by the W-rich alloy area corresponding to the larger triangle (left side of Fig. 1c), i.e., a heterostructure of W-rich and Mo-rich Mo_1−x_W_x_S_2_ alloys was successfully formed, as observed for Mo_1−x_W_x_S_2_ grown on a SiO_2_ or sapphire substrate^11^,^12^,^13^,^14^,^15^,^18^. Structural models for Fig. 1c,a are schematically shown in Fig. 1d,e, respectively.

The heterojunction interface was formed parallel to the edge of the triangular area. An atomically sharp heterojunction interface is clearly visible in the close-up view of the heterojunction interface shown in Fig. 1c. Such a sharp in-plane compositional variation is considered to be a result of the CVD growth sequence. At the initial growth stage, Mo-rich Mo_1−x_W_x_S_2_ alloy was formed in the inner triangle by the Mo rich atmosphere around the substrate owing to the high vapor pressure of MoO_3_ compared with that of WO_3_. Subsequently, the W-rich structure was epitaxially grown from the edge of the Mo-rich structure because of the shortage of Mo and the reduced diffusion of Mo atoms. Similar sequential atomic growth has been observed in previous studies^11^,^18^. Although local fluctuation was observed, the compositional distributions of Mo and W atoms, i.e., the ratio between them, were almost the same over the island and among the islands formed in this sample.

Figure 1f,g show the bias dependence of the Mo_1−x_W_x_S_2_ structure. The STM image is almost flat at a sample bias voltage of *V*_s_ = +1.6 V, while darker W atoms marked by red circles appeared at *V*_s_ = +1.3 V, as observed in Fig. 1c. This is in good agreement with the theoretical result that the local density of states near the conduction band edge *E*_CBM_ is localized at the Mo sites^17^. To observe the electronic structures in more detail, STS measurements were carried out over the W and Mo areas indicated by the white (upper left) and blue squares in Fig. 1g, respectively, and the results are shown in Fig. 1h. Figure 1i shows a magnification of the spectra in Fig. 1h near *E*_CBM_. The spectrum denoted by ‘All’ was obtained by averaging over the surface in Fig. 1g. Although the valence band edges *E*_VBM_ of the three spectra were located close to each other, *E*_CBM_ in the Mo area was about 75 meV lower than that in the W area. From the spectrum ‘All’, the band gap in the area was estimated to be ~2.5 eV, which is between those for pure MoS_2_ (2.40 eV)^20^ and pure WS_2_ (2.73 eV)^21^. Here, *E*_CBM_ and *E*_VBM_ were determined using the bias voltages at which the signal became higher than the noise level. The shift of 75 meV was estimated from the shift between the spectra obtained in the Mo and W areas, respectively, at higher bias voltages of 0.7–1.0 V.

In the same manner as in the estimation of composition-dependent photoluminescence (PL) peak energies^16^, the band gap *E*_g_ of the Mo_1−x_W_x_S_2_ alloy was estimated using the following equation, where b is the bowing factor;
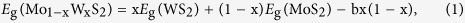


Figure 1j shows a high-resolution STM image of the larger Mo-rich area, in which the compositional ratio of W was estimated to be x = 0.3. The bowing factor of b = 0.14 eV given in ref. 16 was employed. Then, *E*_g_ (Mo_1−x_W_x_S_2_) was macroscopically evaluated to be 2.47 eV, which is in good agreement with the experimental value of 2.5 eV obtained by averaging the STS local spectra over the surface.

### Localized electronic states of the conduction band

**Figure 2:**
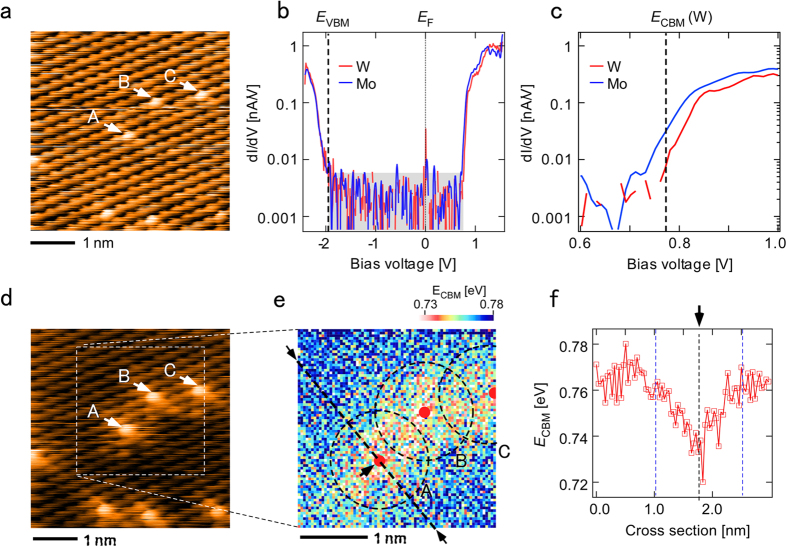
STM/STS on single Mo atoms. (**a**) STM image of an area with isolated single Mo atoms in a WS_2_ area (*V*_s_ = +1.5 V, *I*_t_ = 0.1 nA). (**b**) d*I*/d*V* spectra obtained above Mo atom labeled A in (**a**) and in WS_2_ area. The gray rectangle indicates the noise level. (**c**) Magnification of the spectra in b near *E*_CBM_. (**d**) STM image of the area shown in a obtained at *V*_s_ = +1.2 V. (**e**) Map of the variation in *E*_CBM_ near the Mo atoms A, B, and C in (**d**). (**f**) Cross section along the dashed line in (**e**). The circles in e indicate the area of influence of the Mo-derived state determined from the cross section in (**f**). The red dots in e indicate the positions of Mo atoms.

Next, we analyzed the localized electronic states of the conduction band in more detail using the Mo-derived electronic structure. To obtain an area of isolated Mo atoms easily, we carried out CVD growth using a lower Mo content (see Method). Figure 2a shows a high-resolution STM image of isolated Mo atoms in a W-rich Mo_1−x_W_x_S_2_ area obtained at *V*_s_ = +1.5 V. Mo atoms are brightly imaged, similarly to in Fig. 1. Figure 2b shows d*I*/d*V* spectra obtained above the Mo atom (blue) labeled by A in Fig. 2a and in the WS_2_ area (red). Figure 2c shows a magnification of the spectra near *E*_CBM_. Since the sample is a monolayer, the measurement is free from tip-induced band bending^22^,^23^. In the WS_2_ area, *E*_CBM_ was located 0.77 V above the Fermi level *E*_F_ and *E*_VBM_ was located 1.95 eV below *E*_F_. Therefore, the band gap of this Mo_1−x_W_x_S_2_ monolayer was estimated to be 2.72 eV, which is comparable to that of a pure WS_2_ monolayer obtained from a two-photon absorption spectrum (2.73 eV)^21^. Since the compositional ratio of Mo atoms on this surface estimated from the STM image was very low (x = 0.025), the band gap of the WS_2_ area of this monolayer is considered to be almost identical to that of the pure WS_2_ monolayer. *E*_VBM_ for the spectrum obtained above the Mo atom was located at an almost identical point to that obtained in the WS_2_ area. *E*_CBM_ for the Mo spectrum is about 50 meV lower than that for the W spectrum.

It is known that the contribution of the metal elements to the valence band electronic state is identical for MoS_2_ and WS_2_, namely, both are *d*_xy_ and *d*_x^2^−y^2^_ orbitals, while the main contribution of the metal to the conduction band electronic states is the *d*_z_^2^ orbital in MoS_2_ but the *d*_xy_, *d*_x_^2^_−y_^2^, and *d*_z_^2^ orbitals in WS_2_^16^,^24^. Because of the identical orbital contributions of W and Mo atoms to the valence band, these states are strongly coupled with each other to delocalize the *E*_VBM_ state, whereas Mo-derived states are localized at *E*_CBM_ because the contribution of the coupling between the Mo *d* orbital and the W *d* orbitals to the conduction band is small. Our experimental results clearly show these characteristics.

To what extent does the Mo-derived localized electronic state affect the neighboring WS_2_ area? Figure 2d shows an STM image of the area shown in Fig. 2a obtained at *V*_s_ = +1.2 V to highlight the Mo-derived electronic state. As expected, some W atoms near the Mo atoms are imaged brighter than those in the WS_2_ area. To show this effect more clearly, the variation in *E*_CBM_ near the Mo atoms labeled A, B, and C in Fig. 2d is mapped in Fig. 2e. The value at each of the 75 × 75 pixel was derived from the onset bias voltage (d*I*/d*V*_onset_ = 0.01 nA/V) of each d*I*/d*V* spectrum. Figure 2f shows the cross section along the dashed line in Fig. 2e. A gradual decrease in *E*_CBM_ toward the Mo atom can be observed in Fig. 2f. The variation in *E*_CBM_ around Mo atoms A, B and C are indicated by dotted circles (1.5 nm diameter) in Fig. 2e. This is the first demonstration of the atomically resolved spatial variation in the localized electronic structure of Mo in Mo_1−x_W_x_S_2_ alloy.

### Spatial variation in the band structure of WS_2_/Mo_1−x_W_x_S_2_ heterojunction

**Figure 3:**
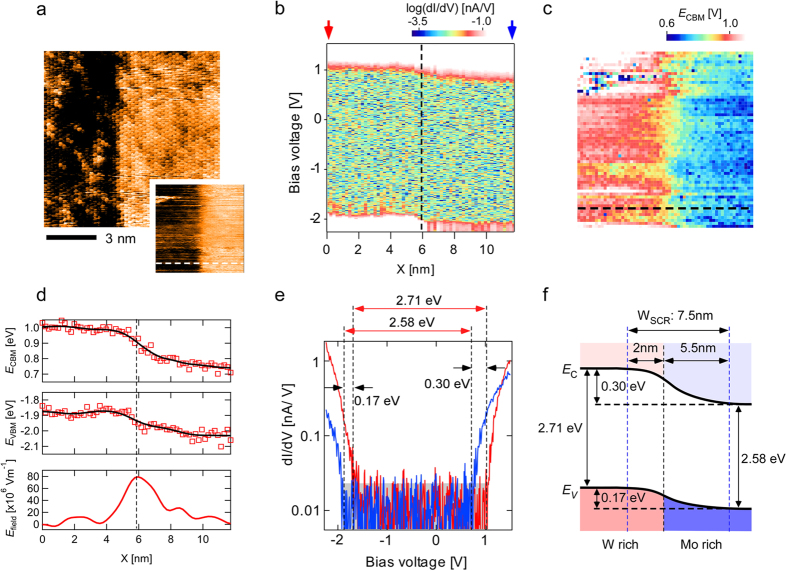
Potential landscape of WS_2_/Mo_1−x_W_x_S_2_ interface. (**a**) STM image of WS_2_/Mo_x_W_1−x_S_2_ interface (*V*_s_ = +1.6 V, *I*_t_ = 0.3 nA). The inset shows the STM image simultaneously obtained with spectroscopy. (**b**) Map of color scale d*I*/d*V* curves calculated from the spatially resolved STS spectra measured along the white dashed line in the inset of (**a**). (**c**) Map of *E*_CBM_ obtained over the area shown in (**a**). (**d**) Cross sections of *E*_CBM_ (top) and *E*_VBM_ (middle), respectively corresponding to the upper and lower edges in (**b**), and electric field *E*_field_ (bottom) obtained by differentiating the cross section of *E*_CBM_ (top). (**e**) d*I*/d*V* spectra obtained at the positions indicated by red and blue arrows in (**b**), which were averaged over the left and right edge lines in the inset of (**a**), respectively. The gray rectangle indicates the noise level. (**f**) Schematic image of the band structure of the WS_2_/Mo_1−x_W_x_S_2_ heterojunction. The values in (**f**) are those in (**e**). W_SCR_ indicates the width of space charge region.

Finally, the band structure of the heterojunction interface was analyzed. Figure 3a shows an STM image of a Mo_1−x_W_x_S_2_-based heterojunction interface similar to that in Fig. 1c, where the left and right regions correspond to W- and Mo-rich Mo_1−x_W_x_S_2_ monolayers, respectively. To investigate the inner potential of the heterojunction, STS was carried out over the surface. The inset in Fig. 3a shows the STM image simultaneously obtained with the STS measurement.

Figure 3b shows a map of color scale d*I*/d*V* curves calculated from the spatially resolved STS spectra measured along the white dashed line in the inset of Fig. 3a. The upper and lower edges of the band gap region, corresponding respectively to *E*_CBM_ and *E*_VBM_, continuously shifted as a function of the distance across the interface, whose position was determined from the STM image (Fig. 3a inset) and is indicated by the dashed black line in Fig. 3b. Figure 3b clearly demonstrates that a type-II staggered gap heterojunction with a nanoscale built-in potential distribution was formed at the interface^9^,^14^,^25^,^26^. This is the first observation of the electronic structure of a TMD heterojunction. The spatial variation of *E*_CBM_ over the surface in Fig. 3a was mapped in Fig. 3c to visualize the electrostatic potential landscape at the interface in more detail, which is almost flat along the direction of the interface compared with the change along the direction crossing the interface (from pink to dark blue).

To better understand the positional relationship between the interface and the electrostatic potential variation, the cross section profile of the *E*_CBM_ map along the dashed line in Fig. 3c (white dotted line in Fig. 3a inset) was plotted (Fig. 3d top) along with the profile of *E*_VBM_ (Fig. 3d middle) along the line at the same position. The comparison of the topographic image with the cross sections of *E*_CBM_ and *E*_VBM_ reveals that the variation in the potential was greater on the Mo-rich side. Namely, *E*_CBM_ is almost constant in the W-rich area, while it gradually changes over the Mo-rich area, possibly reflecting the asymmetric carrier screening length, which may be due to the difference in the doping characteristics and/or dielectric constant between the W-rich and Mo-rich areas. In addition, the profile of the electric field *E*_field_ was obtained from the derivative of the *E*_CBM_ profile with respect to the lateral distance (Fig. 3d bottom). As expected from classical semiconductor theory, the electric field reached its maximum value at the interface position indicated by the dashed line in Fig. 3b,d. The strong electric field of as high as 80 × 10^6^ Vm^−1^ observed at the interface is consistent with the observed charge separation efficiency at the interface^11^.

The red and blue spectra shown in Fig. 3e are the d*I*/d*V* spectra obtained at the positions indicated by red and blue arrows in Fig. 3b, respectively, where neither *E*_CBM_ nor *E*_VBM_ is affected by the built-in potential at the interface. The spectra were averaged over the left and right edges of the inset of Fig. 3c, respectively. From these spectra, the band offsets of *E*_CBM_ and *E*_VBM_ between the W-rich and Mo-rich areas were determined to be 0.30 eV and 0.17 eV, and the band gaps of these areas were also determined to be 2.71 eV and 2.58 eV, respectively. Figure 3f shows the first ever schematic image of the band profile of a WS_2_/Mo_1−x_W_x_S_x_ obtained from the experimental results.

In conclusion, we carried out atomically resolved analysis by low-temperature STM/STS on the electronic structures of a monolayer WS_2_/Mo_1−x_W_x_S_2_ alloy heterojunction that provides a tunable band alignment. The formation of a WS_2_/Mo_1−x_W_x_S_2_ heterojunction on a graphite substrate was confirmed for the first time. Then the atomically modulated spatial variation in the electronic structures, which is the basis for the macroscopic band structure of the WS_2_/Mo_1−x_W_x_S_2_ heterojunction, was directly observed. The macroscopic band structure of Mo_1−x_W_x_S_2_ alloy was reproduced by the STS spectra averaged over the surface. An electric field of as high as 80 × 10^6^ Vm^−1^ was observed at the interface for the alloy with x = 0.3, verifying the efficient separation of photoexcited carriers at the interface. The atomic-scale analysis of TMD heterostructures by STM/STS allows the verification of basic semiconductor physics and is expected to play an essential role in the further advancement of various applications.

## Method

### Sample preparation

Monolayer WS_2_/Mo_1−x_W_x_S_2_ lateral heterojunctions were formed on Kish graphite (Covalent Materials Co.) by high-temperature chemical vapor deposition^28^. The graphite was mechanically exfoliated onto a quartz substrate using Nitto tape (SPV-224). The substrate was placed in a quartz tube (3 cm diameter, 100 cm long) with WO_3_ powder (Aldrich, 99% purity, 100 mg), MoO_3_ powder (Aldrich, 99% purity, 0.2 mg, reduced to 0.1 mg for the measurement of the effect of a single Mo atom shown in Fig. 2), and sulfur flakes (Aldrich, 99.99% purity, 2 g). The quartz tube was then filled with Ar gas at a flow rate of 100 cm^3^/min. The temperature of the substrate and the WO_3_ and MoO_3_ was gradually increased to the growth temperature (1100 °C) over 60 min using an electrical furnace. When the substrate temperature reached the set value, the sulfur was heated at 200 °C for 15–30 min to supply sulfur vapor to the substrate using another electrical furnace. After the growth, the quartz tube was immediately cooled using an electric fan.

## Additional Information

**How to cite this article**: Yoshida, S. *et al*. Microscopic basis for the band engineering of Mo_1−x_W_x_S_2_-based heterojunction. *Sci. Rep*. **5**, 14808; doi: 10.1038/srep14808 (2015).
